# Thermal Conductivity and Electromagnetic Interference (EMI) Absorbing Properties of Composite Sheets Composed of Dry Processed Core–Shell Structured Fillers and Silicone Polymers

**DOI:** 10.3390/polym12102318

**Published:** 2020-10-10

**Authors:** Hyun-Seok Choi, Ji-Won Park, Kyung-Sub Lee, Sang-Woo Kim, Su-Jeong Suh

**Affiliations:** 1 Advanced Materials Science and Engineering, Sung Kyun Kwan University, Suwon 16419, Korea; 2 R&D Center, SMT Co., Ltd., Suwon 16643, Korea; 3 R&D Center, JB Lab. Coporation, Seoul 08826, Korea; roorouny@gmail.com; 4 R&D Center, Nopion Corporation, Suwon 16336, Korea; kslee@nopion.com; 5 Clean Energy Research Center, Korea Institute of Science and Technology, Seoul 02792, Korea; swkim@kist.re.kr

**Keywords:** dual-functional sheet, thermal conductivity, inter-decoupling ratio, core–shell structured fillers, dielectric breakdown voltage

## Abstract

This paper proposes dual-functional sheets (DFSs) that simultaneously have high thermal conductivity (TC) and electromagnetic interference (EMI) absorbing properties, making them suitable for use in mobile electronics. By adopting a simple but highly efficient dry process for manufacturing core–shell structured fillers (CSSFs) and formulating a close-packed filler composition, the DFSs show high performance, TC of 5.1 W m^−1^ K^−1^, and a −4 dB inter-decoupling ratio (IDR) at a 1 GHz frequency. Especially, the DFSs show a high dielectric breakdown voltage (BDV) of 3 kV mm^−1^, which is beneficial for application in most electronic devices. The DFSs consist of two kinds of CSSFs that are blended in accordance with the close-packing rule, Horsfield’s packing model, and with polydimethylsiloxane (PDMS) polymers. The core materials are soft magnetic Fe-12.5%Cr and Fe-6.5%Si alloy powders of different sizes, and Al_2_O_3_ ceramic powders of a 1-μm diameter are used as the shell material. The high performance of the DFS is supposed to originate from the thick and stable shell layer and the maximized filler loading capability owing to the close-packed structure.

## 1. Introduction

Digital mobile electronics have dramatically evolved since their appearance in the late 1990s. With the evolution of mobile electronics, plenty of subsidiary technical challenges have been solved. Especially, thermal and electromagnetic interference (EMI) issues have become critical hurdles that decide the final product availability for market launches. In many cases, thermal- and EMI-related problems are generated in an electronic device simultaneously [1,2,3,4]. To solve these issues, thermal interface materials (TIMs), EMI shielding materials, and EMI absorbers have been used separately to date. EMI absorbers should be used to reduce the secondary reflection electromagnetic waves that are generated from EMI shielding materials like EMI shield cans or EMI shielding coatings or gaskets. Unlike EMI shielding materials, TIMs and EMI absorbers are generally found in the form of sheets or pads. Nowadays, because there is not enough space to apply TIMs and EMI absorbers separately in recent slim mobile electronic devices, such as smartphones, tablet PCs, or laptop PCs, many researchers have been working on developing solutions to meet the existing industrial needs via using a combined TIM and EMI absorbing material as a single solution [1,5].

Some researchers have attempted to achieve both thermal conductivity (TC) and EMI absorbing properties at the same time by applying a blended filler system composed of thermally conductive ceramic powders and magnetic powders [6]; however, it is difficult to maximize each property with a blended filler system because there is a limit to the filler loading capability. Some researchers have tried to develop core–shell structured fillers (CSSFs) to enhance the TC by reducing the thermal contact resistance, and these attempts have been carried out via conventional wet processes with high temperature reduction reactions [6,7,8]; however, wet processes are not practical for commercialization because of the inherent low productivity, as well as the environmental issues from an industrial perspective. On the contrary, dry processes for manufacturing CSSFs are relatively simple and stable thick shell layers can be formed in a short period of time [9,10].

Dry processes for manufacturing CSSFs have rarely been researched via comparison with wet processes, typically because of a lack of concepts or highly efficient equipment. Henschel mixers and super mixers have been widely used as dry processes for surface coating or the modification of various types of fillers; however, in order to manufacture stable and uniform shell layers of CSSFs, another effective and sophisticated dry process needs to be applied. This is because soft magnetic materials, as core bodies, are usually heavy ferrous alloy metals in general, and aluminum oxide as a shell layer also has a high density. A usual Henschel mixer or super mixer is not powerful enough to circulate heavy metals or ceramic powders from the bottom to the top of a vessel. Therefore, a specially designed impeller structure should be applied, and the revolving speed should also be increased to an extremely high level. Recently, some traditional mixer makers have developed state-of-the-art technologies and equipment for manufacturing metal- and ceramic-based CSSFs.

**Figure 1:**
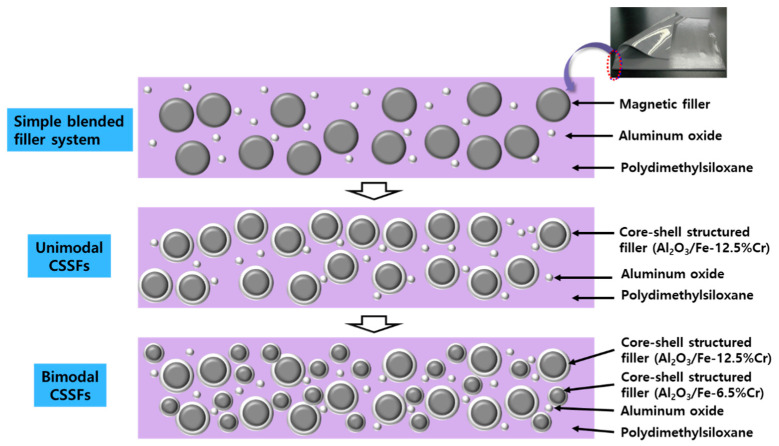
Concepts of three kinds of dual-functional sheets (DFSs) made of simple blended filler systems, unimodal core–shell structured fillers (CSSFs), and bimodal CSSFs.

In this work, two sizes of CSSFs are manufactured by an effective dry process and blended in accordance with Horsfield’s close-packing model in order to achieve TC and EMI absorbing properties at high levels. Some studies of dual-functional sheets (DFSs) have been conducted with unimodal CSSFs; however, in order to achieve the close-packing structure, it is necessary to use a bimodal filler system [11,12]. There have been few reports of using bimodal or trimodal CSSF systems. It is especially rare to find studies about DFSs that feature simultaneous TC and EMI absorbing properties when using bimodal CSSFs. Figure 1 shows concepts of DFSs made of three types of filler systems, namely simple blended filler systems, unimodal fillers, and bimodal fillers.

## 2. Materials and Methods 

**Figure 2:**
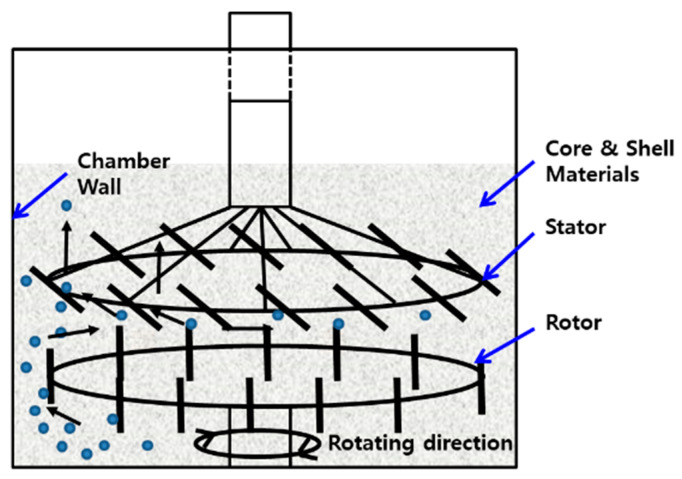
Schematic of the impellor structure of the dry process equipment (COMPOSI, brand name of Nippon Coke & Engineering’s super mixer) and the moving paths of particles.

Spherical Fe-12.5%Cr alloy powders (D_50_ = 50 μm) (410L, Höganäs, Belgium) were used as the core material for large size fillers and semi-spherical Fe-6.5%Si powders (D_50_ = 15 μm) (Mega Flux^®^, Changsung Corp., Incheon, Korea) were used as the core material of small size fillers. The shell material used was aluminum oxide powder (D_50_ = 1 μm) (DAW-01, Denka Company Limited, Omuta, Japan). The core–shell structured fillers (CSSFs) were manufactured by the dry process equipment brand-named COMPOSI (MP5 model, Nippon Coke & Engineering Co. Ltd., Tochigi, Japan). Surface pre-treatment of the Fe-12.5%Cr and Fe-6.5%Si core materials was not needed for forming strongly bonded shell layers due to the high shear stress between the rotor, stator, or chamber wall, where the so-called mechanochemical effect at the surface of the core powders and the surface was highly activated [13,14]. The structure of a rotor and stator in the dry process equipment and the particle moving paths are shown in Figure 2. The core and shell powders were poured into the chamber of the mixer at the same time. The chamber volume was 1.6 L and the quantities of the core and shell powders were 800 and 42 g, respectively. Next, the core and shell powders were milled at the shear rate of 8000 rpm for approximately 8–60 min. The ambient gas was air and the temperature inside the chamber rose to 80 °C during processing.

The particle size distribution (PSD) was analyzed by a particle size analyzer (Cilas920, Malvern Instruments, Malvern, UK). The microstructure and atomic compositions of CSSFs were analyzed by a planar and cross-sectional scanning electron microscope (SEM) with a focused ion beam (FIB) technique (XL-30 FEG, Philips FEI and 3D FEG FEI Quanta, Hillsboro, OR, USA), and additionally a high resolution transmission electron microscope (TEM, Tecnai G2-F20, FEI) and an energy dispersive spectrometer (EDS) (EDAX PV9761, AMETEK, Berwyn, PA, USA).

Two manufactured CSSFs were blended in accordance with Horsfield’s mixing model and were dispersed in addition-curable polydimethylsiloxane (PDMS) polymers (HR-G500A/B, HRS Silicone Co., Ltd., Pyeongtaek, Korea). The compositions were designed with bimodal CSSFs, unimodal CSSFs, and a simply blended filler system, as shown in Table 1. The compounds were dispersed well by a Thinky mixer (ARV-310P, Thinky Corpoaration, Tokyo, Japan) and were then processed to produce sheets of a 3-mm thickness by a typical hot-press method. The curing condition of the PDMS polymers was 150 °C for 30 min. The thermal conductivity (TC) of the cured specimen was measured by a modified transient plane source method (TCi thermal analyzer, C-Therm, Fredericton, NB, Canada) and the inter-decoupling ratio (IDR), as the EMI absorbing property, was evaluated by a network analyzer (PNA 8364A, Keysight, Santa Rosa, CA, USA). The breakdown voltage (BDV) was measured by a Hipot tester (TOS5101, Kikusui, Kobayashi, Japan) at cut-off current of 10 mA. 

## 3. Results and Discussion

### 3.1. Characterization of Core–Shell Structured Fillers (CSSFs)

**Figure 3:**
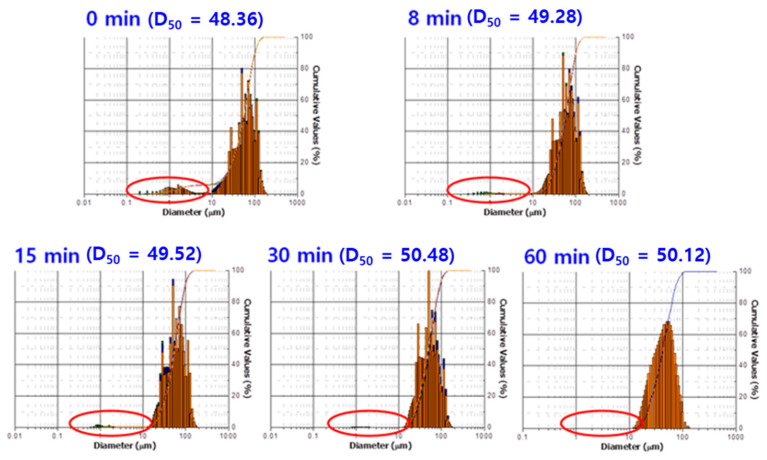
Particle size distribution (PSD) analysis of CSSFs with milling time.

Figure 3 shows the particle size distribution (PSD) analysis results of the Fe-12.5%Cr CSSFs with the milling time. As the milling time increased, the proportion of fine aluminum oxide powders with an average diameter of about 1 μm gradually disappeared. This indicates that most of the aluminum oxide powders are attached onto the Fe-12.5%Cr core surfaces. The small quantity of aluminum oxide residue, shown as a tail in the PSD graphs, can act as a close-packing intermediate and help increase the breakdown voltage (BDV) of the final dual-functional sheets (DFSs). As can be seen in Figure 3, the average diameter value of the CSSFs is not greatly changed because most of the aluminum oxide particles are embedded into the core surfaces and intermixed layers of core and shell materials are formed. These intermixed layers can be observed by a cross-sectional transmission electron microscope (TEM).

**Figure 4:**
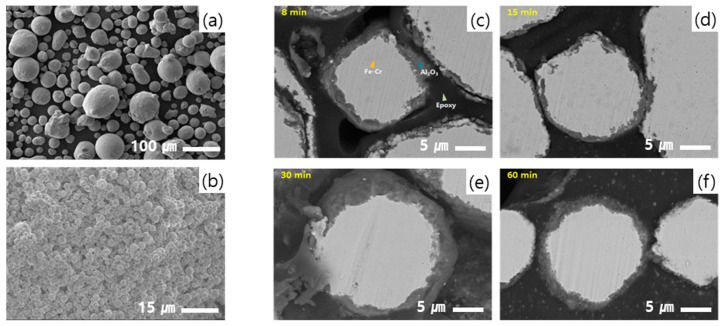
Planar scanning electron microscope (SEM) images of (**a**) Fe-12.5%Cr core powders, (**b**) aluminum oxide shell powders, and (**c**–**f**) cross-sectional SEM back-scattered images of Fe-12.5%Cr CSSFs with milling time.

Figure 4 shows planar images of the Fe-12.5%Cr and Al_2_O_3_ raw materials and cross-sectional images of Fe-12.5%Cr CSSFs with milling time, where the results were found via a scanning electron microscope (SEM) with a focused ion beam (FIB). The thickness of the shell layer increases up until 30 min, and after 30 min the thickness of the shell layer does not change much. It can be considered that once stable shell layers form, additional aluminum oxide powders cannot attach onto the shell surface because the aluminum oxide itself is a stable material and it is not likely that it will bond together via simple mechanical stress alone [15]. There are only hydroxyl (-OH) functional groups on the surfaces of the aluminum oxide particles, and these are not sufficient to act as bonding intermediates. The thickness of the shell layer is approximately ~1–3 μm at the milling time of 30 min, and this is considered to be enough to act as an electrical insulation layer for the BDV property of DFSs. Based on Figure 3 and Figure 4, a milling time of 30 min can be decided as the optimum milling time.

**Figure 5:**
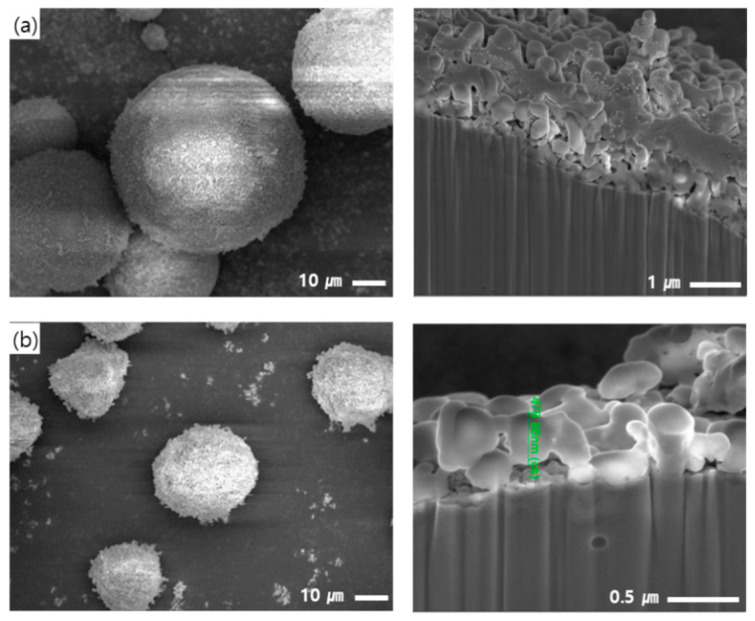
Shapes of (**a**) Fe-12.5%Cr CSSFs and (**b**) Fe-6.5%Si CSSFs milled for 30 min (found via a planar SEM) and the thickness of shell layers (found by a cross-sectional SEM).

The final shapes of Fe-12.5%Cr CSSFs and Fe-6.5%Si CSSFs milled for 30 min by the dry process are shown in Figure 5, displaying planar and cross-sectional SEM images. The surfaces of the CSSFs are reasonably smooth in terms of compounding in PDMS polymers, and the shell layer thicknesses of the Fe-12.5%Cr CSSFs and Fe-6.5%Si CSSFs are 2.5 and 0.5 μm, respectively. The difference of thicknesses between two CSSFs is originated from the difference of average momentum (=mass × velocity) between Fe-12.5%Cr and Fe-6.5%Si particles when they collide with aluminum oxide particles, because their density is 7.78 and 7.48 g/cm^3^ and, accordingly, their average mass (=volume × density) is 5.09 × 10^−8^ and 1.32 × 10^−9^ g, respectively. Each shell layer of the two types of CSSFs has a dense cross-sectional microstructure and can function as an electrical barrier to withstand high voltages.

**Figure 6:**
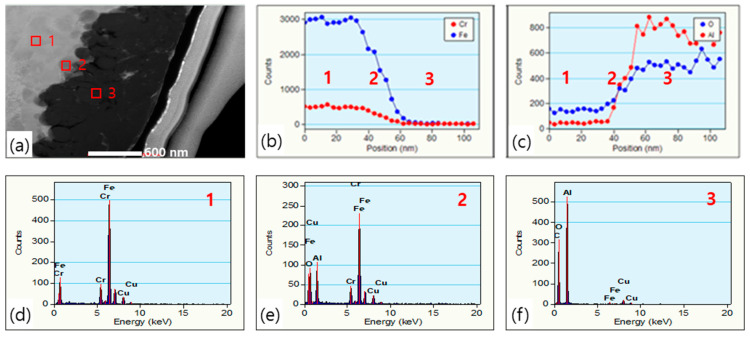
(**a**) Cross-sectional TEM image, (**b**,**c**) EDS line profile of areas 1, 2, 3, and (**d**–**f**) EDS atomic composition analysis of a Fe-12.5%Cr CSSF at areas 1, 2, 3.

**Figure 7:**
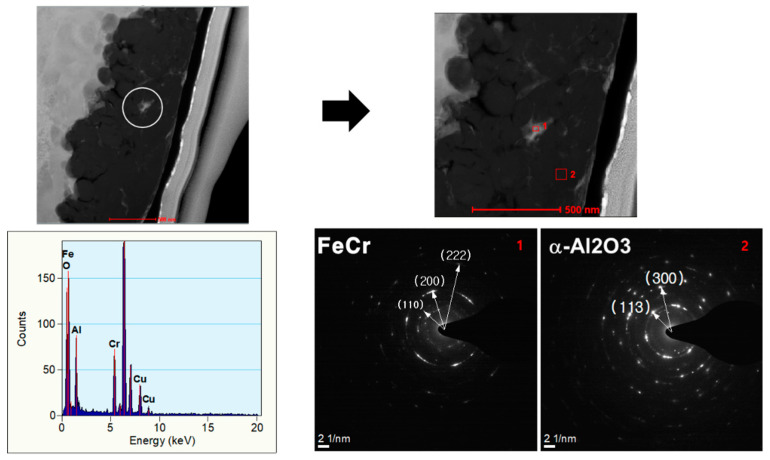
Cross-sectional selected area electron diffraction (SAED) analysis of a Fe-12.5%Cr CSSF.

As shown in Figure 6, the cross-sectional TEM and EDS analysis of Fe-12.5%Cr CSSFs shows that there are areas where atoms from both the core and shell layers coexist. By EDS dark field spot analysis (Figure 6a,d–f) it can be seen that there is an intermixed layer (area 2) and that the core materials are embedded in the shell layers (area 3). A matrix of Al_2_O_3_ shell particles surrounds the core materials in the shell layers. Because of the intermixed layers, a kind of interlocking effect can sustain bonding between the core and shell layers. The thickness of intermixed layers can be analyzed by the EDS line profile method (Figure 6b,c). The thickness of intermixed layers here is about 20–30 nm, which is naturally affected by the original particle size of the shell layers. In the shell layers, small amounts of the core materials (Fe, Cr) were detected by cross-sectional selected area electron diffraction (SAED) analysis (Figure 7). The dim diffraction pattern of Fe-12.5%Cr elements and the bright pattern of Al_2_O_3_ means that there are intermediate Fe-12.5%Cr core materials with the Al_2_O_3_ shell particles. This metallic component could be act as an inorganic binder for interlocking core and shell particles, as confirmed by the high resolution TEM images here.

### 3.2. Characterization of Dual-Functional Sheets (DFSs)

#### 3.2.1. Thermal Conductivity (TC)

**Figure 8:**
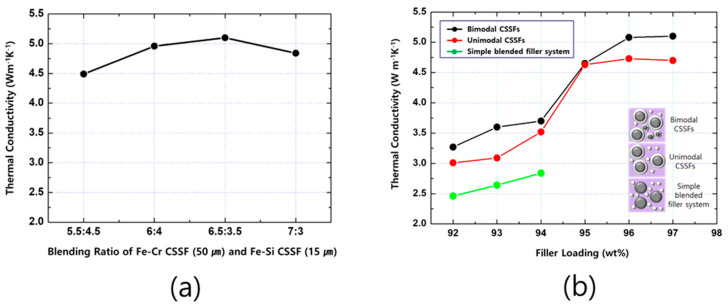
(**a**) Thermal conductivities (TCs) of dual-functional sheets with a blending ratio of larger to smaller sized CSSFs and the (**b**) comparison of the TCs of DFSs made of bimodal CSSFs, unimodal CSSFs, and simple blended filler systems.

Figure 8 shows the TC values of DFSs made from bimodal CSSFs, unimodal CSSFs, and a simple blended filler system. The TC values of the Fe-12.5%Cr and Fe-6.5%Si core materials were 16 and 35 W m^−1^K^−1^ here, respectively. The TC of the pure aluminum oxide was 32 W m^−1^ K^−1^ here. The TC values of DFSs made from bimodal CSSFs were higher than those of DFSs made from unimodal CSSFs or a simple blended filler system. The reason for the high TC of the bimodal CSSFs is the increase in the volume fraction of Fe-6.5%Si (a high TC material), and the total filler loading capability also increases due to the close-packing structure of the two sizes of CSSFs, which is known as Horsfield’s packing model [16,17]. The optimal ratio of a larger sized Fe-12.5%Cr CSSF to a smaller sized Fe-6.5%Si CSSF is 6.5 to 3.5, as can be seen in Figure 8a. At this point, the maximum TC of 5.1 W m^−1^ K^−1^ is achieved and this value is over two times higher than that of the simple blended filler system, as shown in Figure 8b. In the case of a simple blended filler system, the limit of capable filler loading is around 94 wt %; however, in the case of CSSF systems, the filler loading capability increases up to 97 wt %. There are also saturation points for the filler loading capability and TC for the two types of CSSFs. After the filler loading exceeds 96 wt %, the TC values of DFSs made of the two types of CSSFs become saturated, and even the TC of a DFS made of unimodal CSSFs decreases slightly. This phenomenon is mainly related to the dispersion qualities. At an extremely high filler loading content of 97 wt % (82 vol %), compound dispersibility is greatly affected by compounding process parameters, such as batch size, temperature, dispersion time, shear rate, and so forth, and a high-degree of techniques are required to achieve complete dispersion [18]. The dispersion property is evaluated by the porosity of cured DFSs, which is expressed as the difference between the theoretical density (designed density) and the real measured density of the DFSs, as shown in Table 1. In case of unimodal CSSFs, the porosity of a DFS filled with a 97 wt % CSSF is larger than that of a 96 wt % CSSF. Therefore, it is reasonable to assume that the TC decreases in spite of the increased filler loading from 96 to 97 wt % in DFSs made of unimodal CSSFs. In the case of bimodal CSSFs, the TC increases slightly when the filler loading content increases from 96 to 97 wt %. A slight discrepancy in behaviors of varying TC can be detected between the two CSSFs systems. In the case of the unimodal CSSFs system, the saturation point of filler loading for the increase in TC is considered to be at 95 wt %, but in the case of the bimodal CSSFs system, the saturation point is thought to be at 96 wt %, as can be seen in Figure 8b. The actual values of TC in the bimodal CSSFs system at 96 and 97 wt % are 5.08 and 5.10, respectively. Despite the porosity increase, the filler loading increase affects the increase in TC more strongly. Moreover, dispersion capability due to the close-packing structure of the bimodal CSSFs system also has a positive effect on the increase in TC at 97 wt %. The effect of porosity on TC values will be discussed specifically in future works.

When calculated via the equation suggested by Pal et al. [19,20], the theoretical TC values of DFSs made of bimodal CSSFs are much higher than the measured values. It is considered that the reason for the large difference of these TC values is largely because the suggested theoretical models of TC estimation are usually limited to within a low filler loading area of under 60 vol %; however, the volume fraction of the maximum filler loading in this work is over 80 vol % and the deviation of the measured TCs from the theoretical values increases as the filler loading content increases. Therefore, new practical models that contain the effect of the naturally contained porosity during the compounding and sheet-making processes are needed to estimate more accurate TC values with high filler loading contents. This is out of the bounds of this paper and will be discussed in future works.

#### 3.2.2. Electromagnetic Interference (EMI) Absorbing Property

**Figure 9:**
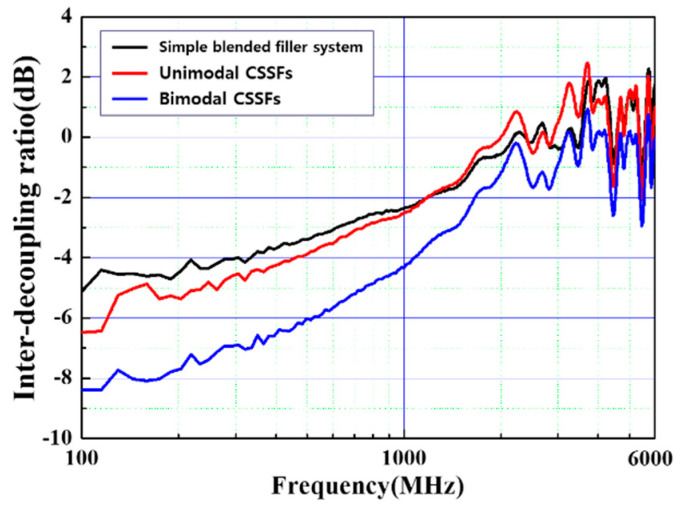
Comparison of the inter-decoupling ratio (IDR) of DFSs made of bimodal CSSFs, unimodal CSSFs, and simple blended filler systems.

In this work, the EMI absorbing property is represented by the inter-decoupling ratio (IDR, *R_de_*), which is measured by a micro-loop antenna method [21]. The IDR is defined as the difference between the transmission S-parameter (*S*_21_) with and without an EMI absorber. The related equation is given as follows:

*S*_21_*(or R_de_)* = *S*_21 *Air*_ – *S*_21 *Sample*_ [dB] (1)

where *S*_21 *Air*_ and *S*_21 *Sample*_ are the S-parameters of a transmitted wave with and without an EMI absorber, respectively. The EMI absorbing property is also improved in the case of DFSs made of bimodal CSSFs when compared with DFSs made of unimodal CSSFs or a simple blended filler system. In Figure 9, at a frequency of 1 GHz, the DFS featuring bimodal CSSFs shows an IDR value of −4 dB, and this value is nearly twice those found with unimodal CSSFs or a simple blended filler system. It is known that the IDR of a material is mainly related to the material’s relative permeability [21,22]. The aluminum oxides in the shell layers have only dielectric permittivity, so the permeability of the CSSFs relies on the magnetic property of the Fe-based core materials. In the case of a Fe-12.5%Cr core material, the initial permeability at a frequency of 1 kHz is 150 and that of Fe-6.5%Si is 100 [23,24]. By a simple average calculation, in spite of decreasing average permeability, the IDR of DFSs made of bimodal CSSFs is higher than that of unimodal CSSFs. It is considered that the close-packed structure of DFSs consisting of bimodal CSSFs shows high density and therefore low transmission. In the process of evaluating a single performance, it is possible to confirm the results that are superior to each evaluation item, but in the case of [25,26] a core-shell structure, to show a maximum of 2.2 W m^−1^ K^−1^ of TC and −23~−40 dB of shielding effectiveness [27].

#### 3.2.3. Dielectric Breakdown Voltage (BDV)

**Figure 10:**
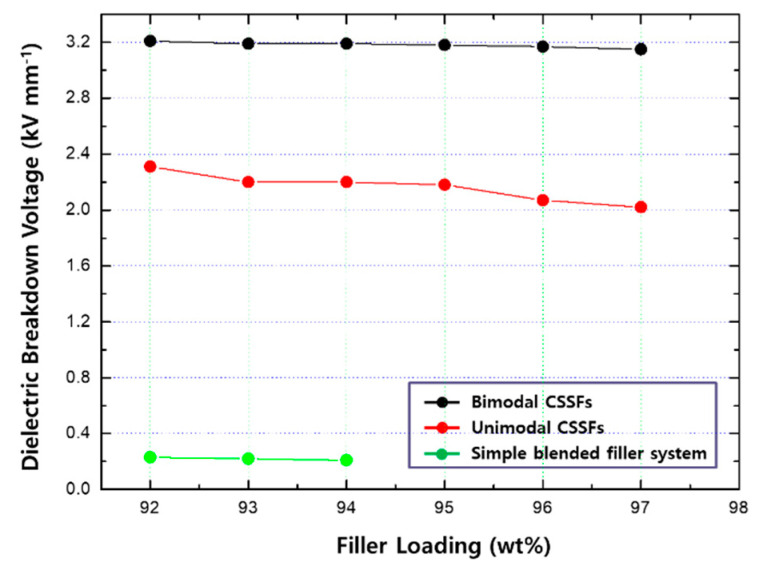
Breakdown voltage (BDV) of DFSs made of bimodal CSSFs, unimodal CSSFs, and simple blended filler systems.

Most metal-cored CSSFs have a weak point of a low BDV for electronic applications because of an incompact shell structure and shallow thickness [28]; however, the densely intermixed shell structure and thick shell thickness of CSSFs manufactured by the dry process in this work enables the DFSs to show high BDV values. In the case of the simple blended filler systems here, the BDV values were measured with an alternating current (AC) at 250 V with a cut-off current of 10 mA. In the case of the unimodal CSSFs and bimodal CSSFs, the BDV values increase up to 2400 and 3200 V (AC) at the same cut-off current for each, respectively. The reasons for the difference of BDVs in the two CSSFs systems are mainly attributed to the porosity effect. The dielectric breakdown voltage of air is known to be 0.4~3.0 kV/mm depending on the conditions, and that of cured polydimethylsiloxane (PDMS) polymer and aluminum oxide is 15 and 14 kV/mm, respectively. The densities of air, PDMS polymer, Fe-12.5%Cr CSSFs and Fe-6.5%Si CSSFs are 0.001, 0.98, 6.91 and 6.00 g/cm^3^, respectively. The densities of the two CSSFs are obtained from geometric calculation. The porosity of the unimodal CSSFs system is 25% larger than that of the bimodal CSSFs system. It means that volume ratio of air in the unimodal CSSFs system is higher than in the bimodal CSSFs system. The density of air is thousands of times smaller than that of PDMS and core-shell fillers, and the effect of air on the BDV is much greater in the unimodal CSSFs system than in the bimodal CSSFs system because the BDV of air is lower compared with other materials. These BDV values of over 1500 V (AC) mean that the DFSs made of CSSFs can be utilized as conventional thermal interface materials (TIMs) and EMI noise suppressors for general electronic applications. Figure 10 shows a comparison of the BDV values between the simple blended filler systems and unimodal and bimodal CSSFs.

## 4. Conclusions

For fabricating core–shell structured fillers, as raw materials for thermally conductive and EMI absorbing dual-functional sheets, a simple but highly efficient dry process that has economic and environmental advantages compared to the conventional wet processes was adopted here. A thermal conductivity of 5.1 W m^−1^ K^−1^ was achieved in the case of the dual-functional sheets made of bimodal core–shell structured fillers, featuring larger sized Fe-12.5%Cr and smaller sized Fe-6.5%Si blended at a ratio of 6.5 to 3.5. The inter-decoupling ratio, as an index of the EMI absorbing property, was measured to be −4 dB at a frequency of 1 GHz here. Especially, by forming a thick and stable shell layer structure for the core–shell structured fillers, the dielectric breakdown voltage was measured to be 3.2 kV mm^−1^ and was much higher than that of the simple blended filler systems here. The three critical properties of high thermal conductivity, EMI absorption, and a high breakdown voltage allow beneficial application of the dual-functional sheets developed here in use with real electronic applications.

## Figures and Tables

**Table 1:** Compositions of three kinds of dual-functional sheets (DFSs) and their porosity values. PDMS: Polydimethylsiloxane.

Sample		Composition (wt %)					Filler wt %	Filler vol %	Porosity ^1^ (%)
		Fe-12.5%Cr	Fe-6.5%Si	Al_2_O_3_	PDMS	Total			
Bimodal CSSFs	BM1	56.81	30.59	4.60	8	100	92	63.27	0.08
	BM2	57.43	30.92	4.65	7	100	93	66.56	0.10
	BM3	58.05	31.25	4.70	6	100	94	70.12	0.09
	BM4	58.66	31.59	4.75	5	100	95	73.99	0.12
	BM5	59.28	31.92	4.80	4	100	96	78.24	0.15
	BM6	59.90	32.25	4.85	3	100	97	82.89	0.28
Unimodal CSSFs	UM1	87.40	-	4.60	8	100	92	62.01	0.13
	UM2	88.35	-	4.65	7	100	93	65.34	0.15
	UM3	89.30	-	4.70	6	100	94	68.92	0.16
	UM4	90.25	-	4.75	5	100	95	72.95	0.24
	UM5	91.20	-	4.80	4	100	96	77.30	0.28
	UM6	92.15	-	4.85	3	100	97	82.11	0.35
Simple blended filler system	SB1	87.40	-	4.60	8	100	92	54.97	0.14
	SB2	88.35	-	4.65	7	100	93	58.11	0.16
	SB3	89.30	-	4.70	6	100	94	61.56	0.23

^1^ Porosity is defined as the difference between theoretically calculated and real measured densities.
